# Effects of active site mutations in haemoglobin I from *Lucina pectinata*: a molecular dynamic study

**DOI:** 10.1080/08927020802144114

**Published:** 2008-08-22

**Authors:** Eunice Ramirez, Anthony Cruz, Diana Rodriguez, Lilen Uchima, Ruth Pietri, Alberto Santana, Juan López-Garriga, Gustavo E. López

**Affiliations:** Department of Chemistry, University of Puerto Rico, Mayagüez, Puerto Rico

**Keywords:** heme pocket, amino acids mutation, computer simulations, *Lucina pectinata*

## Abstract

Haemoglobin I from *Lucina pectinata* is a monomeric protein consisting of 142 amino acids. Its active site contains a peculiar arrangement of phenylalanine residues (PheB10, PheCD1 and PheE11) and a distal Gln at position E7. Active site mutations at positions B10, E7 and E11 were performed in deoxy haemoglobin I (HbI), followed by 10 ns molecular dynamic simulations. The results showed that the mutations induced changes in domains far from the active site producing more flexible structures than the native HbI. Distance analyses revealed that the heme pocket amino acids at positions E7 and B10 are extremely sensitive to any heme pocket residue mutation. The high flexibility observed by the E7 position suggests an important role in the ligand binding kinetics in ferrous HbI, while both positions play a major role in the ligand stabilisation processes. Furthermore, our results showed that E11Phe plays a pivotal role in protein stability.

## 1. Introduction

Haemoglobins and myoglobins have been under intense scientific scrutiny because of their essential role in many important biological systems, and their widespread presence in all organism kingdoms. Although, there are many efforts focused on the understanding of ligand selectivity and stability, many aspects of the ligand binding and unbinding processes observed in these hemeproteins are still under debate. For example, it is well known that in most haemoglobins and myoglobins the ligand binding reaction consists of at least two well separated kinetic processes: bimolecular process and geminate process [1]. Research has also provided some insight into how the haemeoglobin hemepocket micro-environment electronically affects ligand interaction and the effects it has in the ligand binding–unbinding kinetics. It was initially suggested, however, that the size of the distal residue was the main factor controlling ligand-bound structures and ligand binding kinetics, but today it is known that electrostatic forces play a major role. Despite these observations, a combination of factors such as heme pocket steric constrain, hydrogen bonding, local polarity, proximal and globally structural effects are still being proposed in order to explain ligand binding–unbinding kinetics and hence, their selection and stability in vertebrates and non-vertebrates haemoglobins [2].

In this respect, non-vertebrate haemoglobins have been increasingly studied because they can provide information relevant to the evolution of both structural and functional aspects of the globin family [3]. These haemoglobins occur in widely anatomical sites among invertebrates such as the cytoplasm, red blood cells and body fluids, and are believed to bind and transport ligands other than O_2_. They exhibit much wider variations in their primary and quaternary structure [3]; however, the tertiary structure surrounding the heme prosthetic group [Mb fold] is highly conserved among vertebrate as well as non-vertebrate haemoglobins [4]. The Mb fold consists of six to eight α-helical segments connected by short β-turns or loops [5,6], which form a three-on-three helicals and wich, where the heme group binds to the protein through a proximal histidine. It has been proposed that ligand binding kinetics of these non-vertebrate haemoglobins are strongly influenced by the structure of the heme cavity, particularly the size and polarity of residues occupying the distal portion which exerts steric and dielectric effects [5,7].

However, investigations into vertebrate myoglobins and haemoglobin have suggested that ligand binding and unbinding kinetics are not only affected by the size and polarity of the heme pocket residues but also by other residues away from the heme. These heme pocket arrangements provide a ligand stabilisation mechanism with oxygen through a hydrogen bond with histidine [8], since most of vertebrate myoglobins and haemoglobins have a histidine and a leucine in the distal positions E7 and B10, respectively. In various non-vertebrate haemoglobins, the E7 and B10 positions are usually occupied by glutamine and tyrosine resulting in a tight cage for oxygen, which exhibits higher binding affinities relative to vertebrate myoglobins [3]. A hydrogen bond formation between oxygen and the TyrB10 and GlnE7 is thought to be responsible for the ligand stabilisation in these haemoglobins. Several studies have been performed with vertebrate myoglobins [9, 10] to achieve the high oxygen affinities observed in non-vertebrate haemoglobins. Double and triple mutants of sperm whale myoglobins have been studied (ArgCD3Asp, HisE7Val, ThrE10Arg [9]; LeuB10Tyr, HisE7Gln, ThrE10Arg [10]) showing correct alterations in the active site, but these mutations did not reproduce the ligand binding kinetics properties of wild type non-vertebrate myoglobins and haemoglobins. The difference in tertiary structure between mammalian myoglobins and non-vertebrate globins is proposed as an explanation for the low degree of success in these studies, due to the differences in the primary sequence conservation (and no phylogenetic relationship) [3]. Ishikawa et al. [1] demonstrated that the ligand binding–unbinding kinetics are not only affected by the amino acids in the heme pocket moiety, but by residues out of the active site centre.

In order to evaluate ligand selection and stability in hemeproteins, we have performed molecular dynamic simulations using an invertebrate haemoglobin from *Lucina pectinata* as a model system in view of its unique properties. This *clam* contains three different haemoglobins: haemoglobin I (HbI), haemoglobin II (HbII) and haemoglobin III (HbIII). Each haemoglobin has a different biological function, as well as different physical–chemical properties. For example, HbI is a monomeric protein of 142 amino acids residues [11], which exhibits the classical Mb fold [12]. The distal residues PheB10, PheCD1, GlnE7 and PheE11 comprise the HbI heme pocket [13], which is considered a natural occurring mutant haemoglobin [14] compared to mammalian haemoglobins. These amino acid residues form an array of nearly parallel aromatic residues near the heme. In its ferric state, HbI has an extraordinary affinity for hydrogen sulfide (H_2_S) and is also capable of binding and forming stable complexes with nitric oxide, azide (N_3_) and cyanide in this oxidation state. Deoxy HbI has one of the fastest combination rates with oxygen [O_2_] among globins [11]. The ligand binding properties of HbI may be influenced by the low distal pocket polarity and the aromatic nature resulting from this array of phenylalanyl and aromatic residues. This particular arrangement of residues at the distal ligand-binding site is unusual for haemoglobin and has not been observed before in the globin family [13]. Hence, a comparative analysis between of the native HbI and various HbI mutated systems based on computational techniques will yield relevant information concerning the relative structural changes of HbI and how these changes might affect ligand binding properties and selectivity. Specifically, the study intends to provide a molecular description of the effect of active site mutations in the deoxy species, which preludes the ligand migration and binding in the haemoglobin. The results presented here suggest that PheE11 may be involved in structural stability, as well as ligand diffusion through the protein while both GlnE7 and PheB10 are responsible for ligand binding and stability in the HbI heme moiety.

## 2. Methods

**Figure 1:**
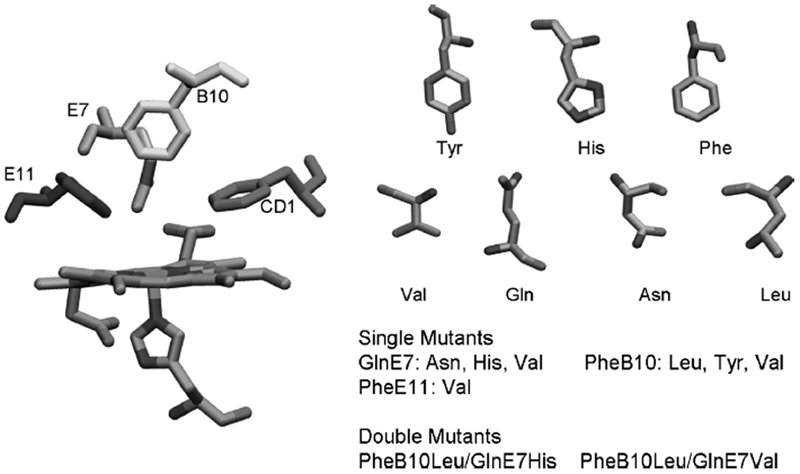
Haemoglobin I heme pocket amino acid arrangement, mutated residue chains and performed mutations.

The systems modelled consisted of one deoxy HbI molecule and 6000 water molecules with the protein structure and coordinates downloaded from the Protein Data Bank (PDB code 1B0B,[15]). The native protein secondary structure was composed mainly by eight helical domains: helices A (3–18), B (20–35), C (36–42), D (52–57), E(58–77), F (88–98), G (103–120) and H (121–140) [17]. The software package Deep Viewer [16] was used to generate the HbI mutations at the heme pocket moiety of ferrous HbI. After the selected residue was mutateda rotation (torsion) of the side chain dihedral angle was performed to obtain the conformation in which the van der Waal interactions were minimised. Figure 1 shows the heme pocket residues and the heme prosthetic group with the amino acids labelled by the helical domain position. The CD1 position refers to the first residue in comprising the loop between helices C and D. The position E7, where the native HbI has a glutamine, was single mutated by a histidine, aspargine and valine. The B10 position was single mutated by a leucine, tyrosin and valine, and position E11 by a valine. Two double mutated systems were studied with mutations at positions B10 and E7, and B10 and E11. Position B10 was mutated by leucine in both systems, while position E11 was mutated with valine and position E7 with histidine. The studied mutations were selected based on previous studies [18] of directed mutagenesis performed in HbI and focused on providing the system with variations in the length, type, and polarity of the amino acid residues, as could be observed in Figure 1.

**Figure 2:**
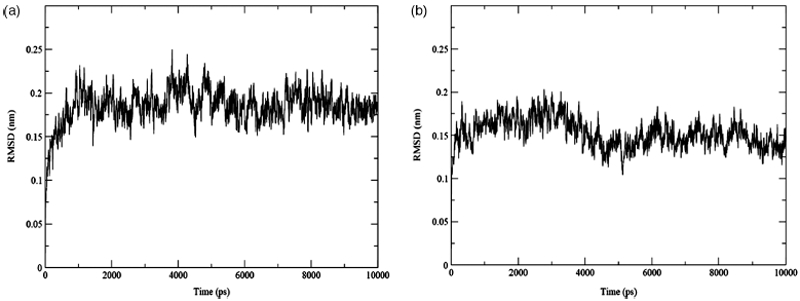
Trajectory RMSD plots of the GlnE7His (a) and PheB10Leu/GlnE7His (b) systems.

All simulations were performed using the GROMACS simulation package version 3.2.1 [19]. The GROMOS96 43a1 force field [20] was used to model the intramolecular protein interactions and the intermolecular interactions between the protein and water molecules. The Single Point Charge (SPC) water model was used to describe the solvent molecules [21]. The HbI molecule was placed in a cubic box with periodic boundary conditions in all directions and solvation was performed using the standard procedure of the GROMACS package. A minimum distance between the protein and its periodic image was set to 2nm to prevent the interactions between them. Initially the energy of each system was minimised with the steepest descent algorithm and 600 ps of position restrain dynamics. The final configuration of this procedure was used as starting point for the production MD runs. The simulations were carried out in a constant temperature, pressure and number of molecules ensemble with a temperature of 298 K. A Berendsen thermostat with a coupling constant of 0.1 ps was used to maintain constant temperature, while the pressure was isotropically maintained constant by coupling it to a Berendsen barostat at 1atm, [22]. Long electrostatic interactions were calculated using the particle mesh Ewald algorithm [23] and a twin range cut-off of 0.9/1.4 nm was applied for van der Waals interactions. Neighbour lists were utilised and updated every fifth integration step. All protein and water bond lengths were constrained using the LINCS and SETTLE algorithm, respectively [24,25]. The simulations were run for 10 ns, which was enough time to obtain proper convergence of the computed properties. The time step for all the simulations was set to 2fs. Initially, the data analysis was performed using different average structures with variable numbers of frames. However, it was found that the best average structure was obtained when the last 2ns of the simulation were used. All the trajectory analyses were performed using GROMACS, while the molecular graphic images were generated using visual molecular dynamics software [26]. Root mean square deviation (RMSD) analyses were performed to evaluate proper convergence of the system. The RMSD was evaluated during the whole trajectory by comparing each frame with the initial configuration, and the average values of RMSD were calculated for the last 2ns of the trajectory. Figure 2 shows the evolution of the Cα atoms RMSD values with respect to the crystal structure for GlnE7His (panel A) and PheB10Leu/GlnE7His (panel B) systems, where the convergence of the systems can be observed owing the RMSD small fluctuation during the last 2ns [27]. Similar results were obtained in the other mutated systems.

To investigate the overall structural behaviour of the native and mutated HbI systems, the molecular dynamic simulations were analysed based on the radius of gyration (Rg), hydrophobic/hydrophilic/total solvent accessibility surface area (SASA), secondary structure evolution (SSE), distances analysis and root mean square fluctuations (RMSF). The RMSD analysis was also used to describe the flexibility of the systems. The last 2ns of the simulations were used to create a structure representing the average position of all the atoms. This structure was used to calculate Rg, SSE, SASA, RMSF and distances analyses of the systems. For each of the structural properties mentioned a single value for each property was computed and reported as the average value. These structural properties were analysed for the whole protein (all the atoms) and specific protein secondary structure domains (Cα atoms), that were select based on the changes in the average structure of the native HbI after the 10 ns simulation. Time line diagrams were generated to determine the time evolution of the protein secondary structure.

## 3. Results and discussion

### 3.1 Overall structural analysis

**Figure 3:**
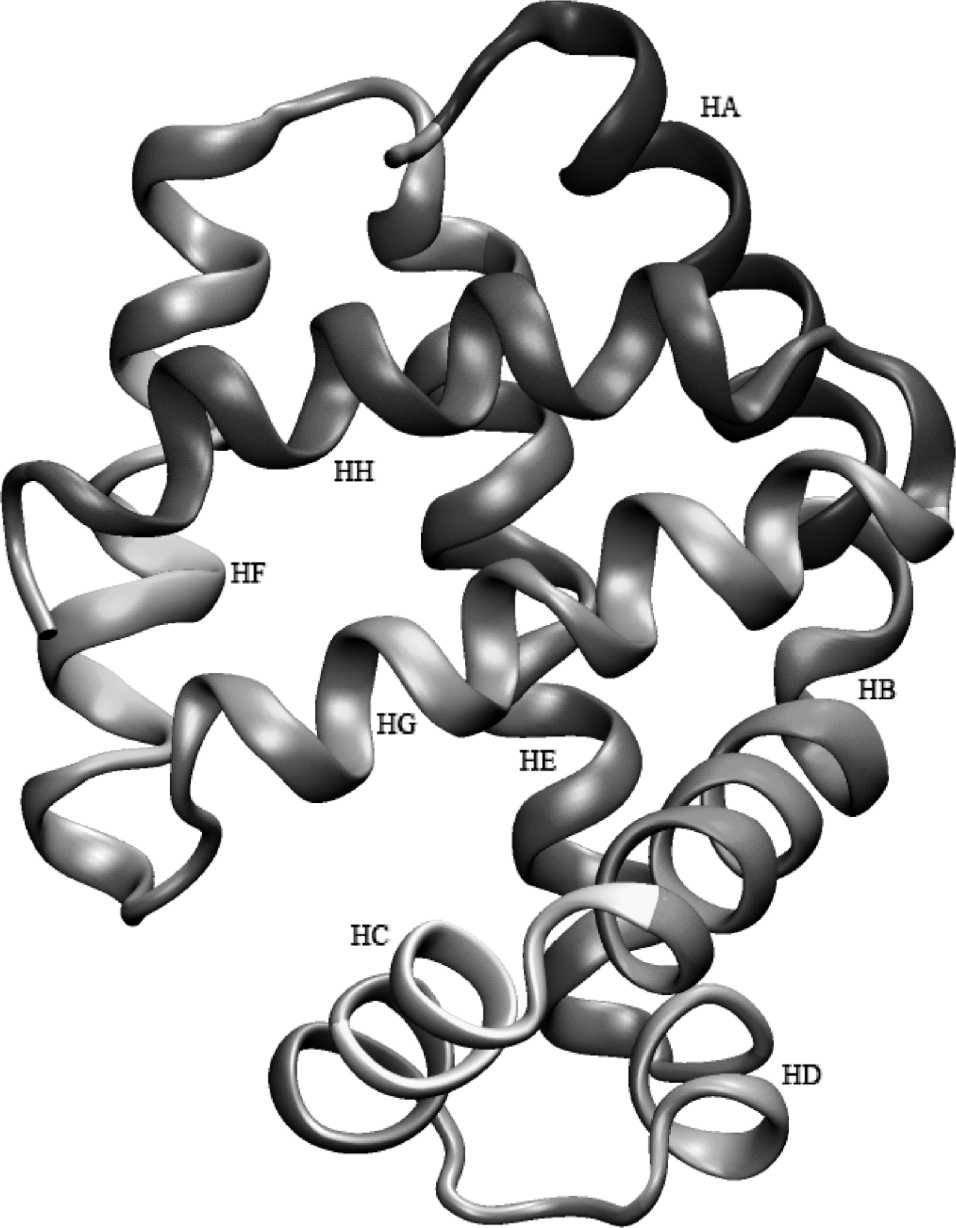
Native haemoglobin Isimulated structure. The amino acid helical sequence is colour as follow: HA (3-18-blue), HB (20-35 – red), HC (36-42 – yellow), HD (52-57 – orange), HE (58-77 – tan), HF (88-98 – green), HG (103-120 – cyan) and HH (121- 140 – purple).

Figure 3 shows the averaged structure of native HbI after a 10ns simulation, where the amino acids described by Torres-Mercado et al. [16] as comprising the molecule helical domains are labelled and highlighted to simplify the structural analysis. These helical domains are represented as follows: the residues in the segments HA (3–18– blue), HB (20–35–red), HC (36-42–yellow), HD (52-57– orange), HE (58-77–tan), HF (88-98- green), HG (103- 120 -cyan) and HH (121–140 – purple). The random coil or turn segments can be observed in Figure 3 coloured in silver, as well as some residues that after the simulation became part of helices. The secondary structure domains assigned to the systems in the SSE structural analysis represent the conformation that the amino acid residues predominantly adopted during the last 2ns of the simulation. As mentioned above, after the 10ns simulation of the native HbI several residues initially comprised in random coil or β-turn conformations became part of helical domains; specifically, the residue sequences 43-48, 78-79, 82-87 and 99–100. Also, it was observed that a 3–10-helix [117–121] changed to form a regular α-helix.

The RMSD showed no significant fluctuations around its average value during the last nanosecond of the simulations. Hence, as explained in the literature [27] oscillation arounda constant value of the RMSD implies that the system has reached *stable or metastable states*. The global RMSD analysis showed that the systems mutated with valine (GlnE7Val and PheE11Val), including the double mutated system PheB10Leu/PheE11Val, exhibited a higher degree of flexibility compared to the other systems with RMSD values of 2.5, 2.9 and 2.8 Å, respectively. The RMSD analysis by secondary structure revealed that helices A–D and the β-turn EF (Figure 3) were more flexible in the mutated systems than in the native protein. As will be discussed later, no significant secondary structure changes were observed in the HA and HB domains: hence the high RMSD observed is assigned to a whole helical domain displacement (translation). On the other hand, the overall Rg and SASA analyses showed only slight differences between the native HbI and the mutated systems. Specifically, the native HbI had a lower Rg compared to the mutated systems, with differences not exceeding 5%. In all cases, when the native structure was compared to the mutated systems, the total hydrophilic SASA values increased between 2 and 5%. Helices A-D showed increments in their hydrophilic SASA and adecrease in their hydrophobic SASA. The randomcoil or β-turn domains showed small changes in their hydrophobic/hydrophilic SASA, except for the CD segment, which exhibited a tendency to decrease its hydrophobic SASA. Hence, we suggest that the increment in SASA corresponds primarily to an increase in the exposure of the protein hydrophilic portions to the solvent. In general, single or double mutations induced a slight increase in the SASA, particularly for the systems involving valine mutations at position E11.

Table 1 summarises some secondary structure information of the systems. Columns one and two correspond to the system and protein sequence, respectively. The secondary structure assigned to the simulated systems is described in columns three and four, where H, T and R represent the α-helical, β-turn and random coil domains, respectively. Column three shows the native HbI secondary structure after completing the simulation, while column four illustrates the structural domain assignment to the mutated systems. Column five depicts the segment unfolding degree and column six describes the affected domain. For example, the PheB10 Leu system exhibited changes in the residues 47-48, which after the simulation was performed, were assigned to a α-helical domain in the native HbI while in the mutated molecule were part of a random coil domain. As it is shown in Table 1, the main effects caused by the mutations were conformational changes on helices C and D, and the loop between these helices (CD, Figure 3). The single mutations PheB10Leu, GlnE7His and GlnE7Asn had small effects in the secondary structure domain, where only the unfolding of two residues near the C helix were observed. However, the single mutated systems PheB10Tyr, PheB10Val, GlnE7Val and PheE11Val, as well as the double mutated systems, induced major changes in the D helix domain. Basically, all but GlnE7Val lead to thetotal unfolding of this domain. Furthermore, the mutations involving the E11 position not only induced total unfolding of the D helix but a certain degree of unfolding of the C helix as well. The GlnE7Val mutation also affected the C helix but not as significantly as the E11 mutations. The generalised effect induced by the E11 mutation seems to decrease with the additional mutation of a PheB10 by a leucine, where the extent of unfolding observed in the C helix segment is smaller in the double mutated PheB10Leu/PheE11Val system than in the single mutated system.

**Table 1:** Summary of domains unfolding degree of the mutated systems after a simulation was performed.

		Secondary structure details			
				
Mutated system	Sequence	Native	Mutant	Unfolding degree	Affected domain
B10Leu	47–48	HH	RR	–	CD
B10Tyr	48	H	T	–	CD
	52–57	HHHHHH	TTTTRR	Total	HD
B10Val	43–48	HHHHHH	RTTTTT	–	CD
	52–58	HHHHHHH	TTTRTTT	Total	HD
	97–100	HHHH	TTTT	Parcial	HF/FG
E7His	47–48	HH	RR	–	CD
E7Asn	47–48	HH	RR	–	CD
E7Val	42–48	HHHHHHH	RRRRRRT	Parcial	HC/CD
	56–58	HHH	RRR	Parcial	HD
E11Val	36–48	HHHHHHHHHHHHH	TTTTTTTTTTTTR	Total	HC/CD
	52–57	HHHHHH	TTTRRR	Total	HD
B10LeuE7His	47	H	T	–	CD
	52–57	HHHHHH	TTTTTR	Total	HD
B10LeuE11Val	39–48	HHHHHHHHHH	TTTTTTTTTT	Parcial	HC/CD
	52–58	HHHHHHH	TTTTTTT	Total	HD

The sequence (column2) secondary structure is represented by H, Rand T (columns 3 and 4), which represents the α-helical, random coil, and β-turn domains, respectively. The CD domain represents the loop that connects the C and D helices.

**Figure 4:**
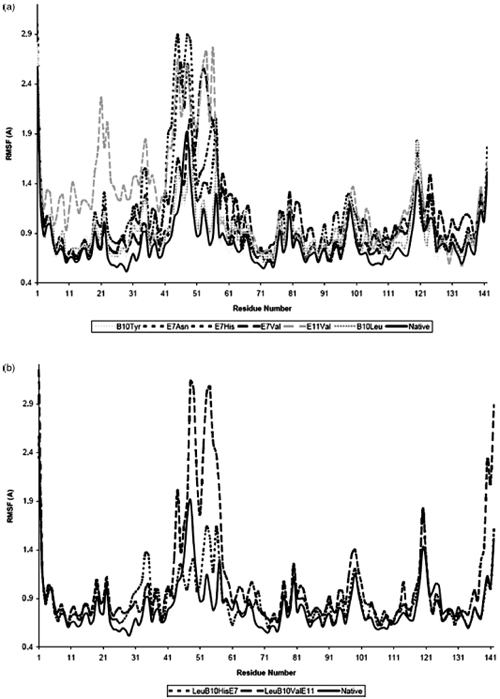
Difference between the mutated RMSF values minus the native RMSF values for the single mutated (a) and double mutated (b) systems.

To further investigate secondary structural changes upon protein mutation, RMSF analyses were performed in native HbI, as well as the mutated systems. Figure 4 shows the difference between the mutant HbI RMSF and the native RMSF for the single mutated (a) and double mutated (b) systems. Interestingly, only the mobility of the chains in the 30–60region showed a significant increment upon mutation. The PheE11Val system again exhibited the higher degree of side chains flexibility, which extends to residues 10–57. However, not all these residues were involved in the unfolding process observed in this system. Similar results were obtained with GlnE7Asn, which showed high flexibility in the side chains 42–48, but exhibited secondary structure changes involving only residues 47–48. Hence, we suggest that the side chain rearrangements induced by the mutations are not necessarily related to adirect change in the secondary structure of the residue, but to structure displacements that change the residue–residue and/or residue–solvent interactions.

Table 2 summarises the average distance between the Cα of the mutated residue and the Cα of all the residues in the affected protein segment exhibitinga major change in the secondary structure domains (Table 1). For example, the segment 43–48 was affected by the PheB10Val; thus, we measured the distance from the Cα of Val28 to the Cα for all the residues comprising the segment [Phe43, Ser44, Gly45, Leu46, Phe47 and Ser48]. After these distances were measured an average value was calculated, which is reported in Table 2. Our results showed that a residue change in the heme pocket moiety affected notonly the environment close to the residue, but also the secondary structures (Table 1) as far as 16.4 Å away from the altered position. However, the mutations appear not to affect significantly the secondary structure domains close to the heme pocket. The GlnE7Asn and GlnE7His induced secondary structural changes as much as 11.7 and 11.9 Å away from the active site positions, respectively. Similarly, the PheB10Val and PheB10Leu affected the secondary structure as far as 10.9 and 11.1 Å, respectively. In general, the most dramatic effect was observed when the PheE11 was changed, leading to secondary structural changes 15–16.4 Å away from the E11 position. The PheB10Leu/GlnE7His and PheB10Leu/PheE11Val systems affected segments on an average distance from the B10 and E7 positions of 10.5 to 14.5 and 13.1 to 15.3 Å, respectively. Interestingly, in the GlnE7His system its range of effects increased in the PheB10/GlnE7His system, while in the case of the PheE11Val the range of effect decreased in the PheB10Leu/PheE11Val. Hence, it cannot be established how the effect of individual alterations are combined in a double mutated system, because the GlnE7His and PheE11Val do not have the same effect on residues in the studied systems.

**Table 2:** Average distance from the Cα of the mutated amino acid residue to the Cα of certain residues comprising the affected protein domain.

System	Distance (Å)
B10Tyr	9.78
B10Leu	11.1
B10Val	10.9
E7Asn	11.7
E7Val	13.6
E7His	11.9
PheE11Val	16.4
B10LeuE7His	10.5/14.5
B10LeuPheE11Val	13.1/15.3

**Figure 5:**
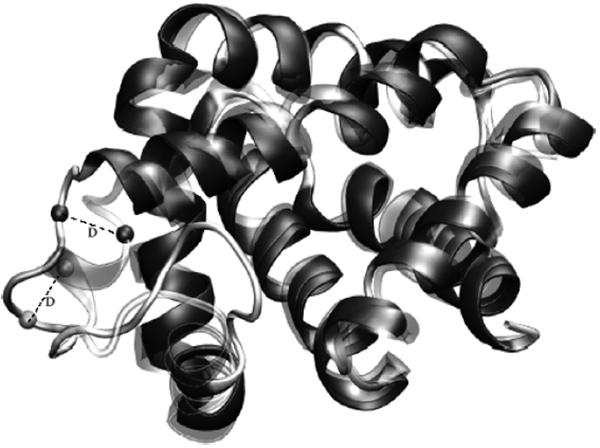
Superposition of native HbI and a mutated system structures. Identical α-carbons in both sequences are marked to show how the distances (D) between systems were measured.

To understand the displacement effect of the mutations in the affected segments, the distances between the simulated native HbI molecule and the mutated systems were measured. Figure 5 shows a superposition of the simulated native HbI (light gray) and the mutated protein (dark gray) where the displacements mentioned can be easily observed. The spheres represent the Cα of specific residues in the primary sequence; i.e. the Cα of Gly45 in the native and mutated system. The distance measurement was performed always between the same residue in both molecules. The distance analyses revealed that the systems mutated with Asn, Leu and His showed changes smaller than 3Å, thus exhibiting no significant displacement from the native HbI. On the other hand, the valine mutations showed changes up to 9.1 Å relative to the native structure.

These overall structural analyses contribute to the hypothesis drawn from other molecular dynamic simulations [28], that protein perturbation by some mutations provokes conformational fluctuation and displacement, which can modulate protein dynamics and ligand diffusion through the haemeproteins. Furthermore, it has been reported that helical domain displacements in human haemoglobin of about 3 Å could change the protein activity, as well as ligand kinetic properties [29]. These findings strongly suggest a pivotal role of the E11Phe benzyl side chain in the overall HbI structural stability that can in turn affect ligand internal migration into the native protein matrix. In this respect, it has been suggested recently that the position of the E11 side chain in myoglobin regulates ligand internal movement through the interior of the protein [30]. It was observed in that study that replacement of the naturally occurring E11Val by Phe decreases the dissociation constant due to a higher extend in ligand geminate rebinding. The nearly 100% geminate rebinding was attributed to the aromatic side chain, which occupies a fixed position that hinders ligand access from the distal pocket to the interior of the protein. A similar mechanism can be invoked here since a five fold decrease in the O_2_dissociation rate constant is observed for the native HbI, which bears Phe at position E11, when compared with a PheE11Val HbI mutant (325 s^−1^ for the PheE11 Val mutant and 61s^−1^ for native HbI)(R. Leon unpublished results 2005). Hence, we suggest that the aromatic benzyl side chain is not only important in protein structural stabilisation but that it dictates ligand migration through the HbI protein matrix as well.

### 3.2 Distal cavity fluctuation

**Figure 6:**
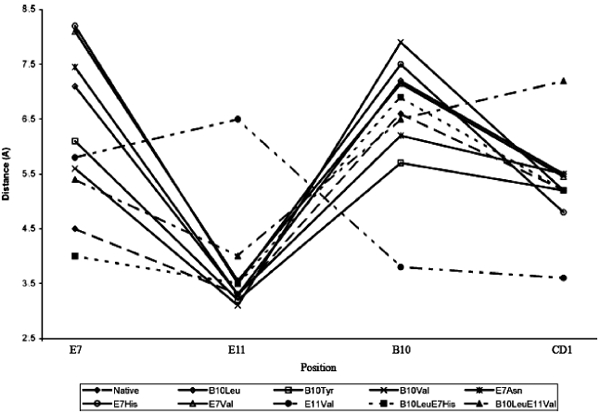
Distance between heme iron and heme pocket residues at positions E7, E11, B10 and CD1 for each mutated system.

To evaluate the effect of the changes in the heme pocket moiety, the distances between the heme pocket residues and the heme iron were measured for each mutated system. Figure 6 plots the distance between heme iron and the heme pocket amino acid residues in positions E7, E11, B10 and CD1. Positions E11 and CD1 did not change dramatically upon active site mutation except in the systems involving the E11 position. The average distance fluctuations were 3.4 and 1.1 Å for the E11 position, and 1.5 and 2Å for the CD1 position in the PheE11 Val and PheB10Leu/PheE11 Val, respectively. Displacement of CD1Phe has been observed in various ferrous myoglobin systems due to a simultaneous movement of the distal residue E7 and the porphyrin macrocycle in the presence of an external ligand [31,32]. It has been shown recently [34] that upon ligand photodissociation the doming of the heme towards a deoxy configuration, as well as the relaxation of the residue at position E7 provoke a displacement of the CD1Phe away from the heme. Therefore, since no ligand was included in our calculations and the heme macrocycle was already in a deoxy ‘out of plane’ configuration, movement of CD1Phe was not expected here. Nevertheless, recent molecular dynamic simulations of ferric unligated HbI showed the flexibility of CD1Phe to be influenced by the opening of the E7Gln gate [33]. Their results suggested that when the E7Gln was in its closed conformation CD1Phe moves towards the Fe^III^ atom. However, as Figure 6 shows, movement of the residues at the E7 position away or toward the ferrous iron did not provoke a substantial displacement of the CD1Phe. As mentioned above, movement of CD1Phe in myoglobin is induced in part by the configuration of the porphyrin group. Thus, differences in the electronic structure of unligated ferrous and ferric HbI can induce variation in their heme chromophore spin states and hence, their heme configurations, which may account for the variation of CD1Phe flexibility in both systems.

Position B10 and E7 on the other hand, were very sensitive to active site mutations. The mutations PheB10Leu, GlnE7His and GlnE7Val induced displacements exceeding 2.5 Å compared to native HbI. The Phe B10Tyr, PheB10Val and PheE11Val induced displacements in the B10 position of 1.1, 1.2 and 3.0 Å, respectively, compared to native HbI. Hence, these results revealed that the E7 and B10 positions were the most affected upon mutation no matter the mutated position. This behaviour could be observed in Figure 6 where theCα of residues at position B10 and E7 show a fluctuation range of ~4.4 Å, while the E11 and CD1 exhibited a limited range of displacements for most of the systems. Interestingly, these positions have been identified as playing an important role in ligand binding and stabilisation processes. Rizzi et al.[34] suggested that ferric HbI stabilises the H_2_S ligand by means of hydrogen bonding with E7Gln and that this residue exhibited a higher degree of flexibility in comparison to the E7His commonly found in mammalian haemoglobins. This observation led to the proposal that the flexibility of E7Gln in ferric HbI facilitates the exit of water molecules located in the heme pocket, allowing hydrogen sulphide binding to occur. Indeed, recent molecular dynamic simulations of ferric HbI in its unligated state confirm this interpretation [33]. In this study, it was observed that E7Gln interchanges an open and closed configuration within 200ps allowing the entrance and stabilisation of the H_2_S ligand. As Figure 6 shows, the mutations of distal residues at positions B10 and E11 induced large displacements of GlnE7, suggesting a high degree of flexibility for this residue in the deoxy species. Thus, this position might play an important role in ligand binding in the deoxy HbI species, as well as in the ferric one. These results are consistent with the hypothesis drawn from infrared experimental data, in which a flexible E7Gln was observed for both ferric and ferrous HbI complexes [35]. Moreover, in accordance with our molecular dynamic results, it is also suggested that not only E7Gln was involved in ligand reactivity in the HbI ferrous systems, but that Phe in the B10 position was also directly involved. This interpretation is further confirmed by analysing the binding kinetics of oxygen with various HbI mutated systems [35]. For instance, our work demonstrates that replacement of E7Gln by either Asn or Val provoked a displacement of these residues as well as the residue in the B10 position away from the heme iron centre, producing an open distal cavity that may facilitate the entrance of ligands. Indeed, the association rate constants for these HbI mutated systems increases from 190 × 10^6^ M^−1^ s^−1^ for the native protein, to 230 × 10^6^ and 490 × 10^6^ M^−1^ s^−1^ for the GlnE7Asn and GlnE7Val mutants, respectively, indicating that the open cavity observed in our study allows O_2_ to react readily with HbI heme iron centre. Our results also suggest that substitution of B10Phe by Leu induces a distal displacement similar to that observed in GlnE7Asn, while replacement by a polar residue like Tyr in that B10 position has the opposite effect. As Figure 6 shows, in the PheB10Leu mutation an open cavity is produced, while in the PheB10Tyr a closed heme distal site is formed due to the movement of the B10Tyr towards the heme iron centre. Oxygen binding kinetics of these HbI B10 mutants indicate that for the Leu system, the association constant remains practically the same as the GlnE7Asn, while a 30-fold decrease was observed for the Tyr mutant (from 190 × 10^6^ to 6.8 × 10^6^ M^−1^ s^−1^). Hence, these theoretical and experimental results suggest a similar pathway for ligand movement into the distal cavity in the GlnE7Asn and PheB10Leu mutants, and a barrier for ligand binding into the heme iron centre for the PheB10Tyr system due to its movement towards ferrous centre. Taken together, these results demonstrate the direct implication of E7Gln and B10Phe in ligand binding and stability in the ferrous HbI system.

## 4. Conclusion

In this work, we presented molecular dynamic simulations of native HbI and several HbI mutated systems solvated by water. The results were analysed based on the following properties: the SSE to characterised domain changes, RMSD, RMSF, Rg and SASA. Distances between the heme iron to the heme pocket residues, as well as between the heme pocket residues to the residues comprising affected protein domains upon mutation were measured to characterise the deoxy HbI systems. The active site mutations did not affect significantly the secondary structure of domains close to the heme pocket, such as helixes B and E, but had a significant effect on domains far away from the active site. The most affected domains were the C and D helices suffering partial or total unfolding upon certain mutations, enhanced in the mutations including position E11. In addition, RMSF analysis indicates that the PheE11Val system exhibits higher degree of side chains flexibility and segments displacements, which may affect significantly ligand diffusion through the protein matrix as it has been observed before in human haemoglobin. These findings strongly suggest a direct role of residue at the E11 position in the overall structural stability and hence, ligand internal movements in the native HbI protein.

Distal cavity analysis shows a lack of motion of the CD1Phe residue upon active site mutations in ferrous HbI due to absence of both, relaxation of the E7 residue and heme conformational changes, which are produced by the presence of external ligands. The fact that CD1Phe movement was observed in the unligated ferri cHbI species [34] and not in its ferrous counterpart suggests differences in heme configuration in both systems, probably induced by variations of heme chromophore spin states, since CD1Phe displacement has been associated with heme conformational changes. On the other hand, the distance analysis revealed that the most affected heme pocket positions upon mutations were the positions E7 and B10, which exhibited significant changes no matter the mutated position. Based on our results and previous studies [33,34], it is plausible to suggest that the high flexibility of E7Gln is an important factor in the ligand binding kinetics in ferrous HbI while both the B10 and E7 positions play amajor role in the in ligand stabilisation processes. Moreover, our data unambiguously shows that E11Phe plays a pivotal role in protein stability, which modulates ligand diffusion pathways in the ferrous HbI system.
